# Next generation selective estrogen receptor degraders in postmenopausal women with advanced-stage hormone receptors-positive, HER2-negative breast cancer

**DOI:** 10.3389/fonc.2024.1385577

**Published:** 2024-05-10

**Authors:** Baha’ Sharaf, Abdelrahman Hajahjeh, Hira Bani Hani, Hikmat Abdel-Razeq

**Affiliations:** ^1^ Department of Internal Medicine, King Hussein Cancer Center, Amman, Jordan; ^2^ School of Medicine, The University of Jordan, Amman, Jordan

**Keywords:** metastatic breast cancer, endocrine therapy, SERDS, aromatase inhibitors, fulvestrant, elacestrant, ESR1

## Abstract

Breast cancer is the most prevalent malignancy in women, and is characterized by its heterogeneity; exhibiting various subgroups identifiable through molecular biomarkers that also serve as predictive indicators. More than two thirds of breast tumors are classified as luminal with positive hormone receptors (HR), indicating that cancer cells proliferation is promoted by hormones. Endocrine therapies play a vital role in the effective treatment of breast cancer by manipulating the signaling of estrogen receptors (ER), leading to a reduction in cell proliferation and growth rate. Selective estrogen receptor modulators (SERMs), such as tamoxifen and toremifene, function by blocking estrogen’s effects. Aromatase inhibitors (AI), including anastrozole, letrozole and exemestane, suppress estrogen production. On the other hand, selective estrogen receptor degraders (SERDs), like fulvestrant, act by blocking and damaging estrogen receptors. Tamoxifen and AI are widely used both in early- and advanced-stage disease, while fulvestrant is used as a single agent or in combination with other agents like the cyclin-dependent kinase 4 and 6 (CDK4/6) inhibitors (palbociclib, abemaciclib, ribociclib) or alpelisib for advanced-stage disease. Currently, SERDs are recognized as an effective therapeutic approach for the treatment of ER-positive breast cancer, showing proficiency in reducing and blocking ER signaling. This review aims to outline the ongoing development of novel oral SERDs from a practical therapeutic perspective, enhancing our understanding of the mechanisms of action underlying these compounds.

## 1 Introduction

Breast cancer surfaces as a prevalent health issue, influencing a substantial number of women worldwide. According to GLOBOCAN 2020, it is the most commonly diagnosed cancer among women in 185 countries, and is a leading cause of mortality, too (1). The molecular heterogeneity of breast cancer makes it a challenging disease to treat, highlighting the need for active research to develop new drugs that can tackle the different tumor subtypes and at different phases throughout the disease course (2). Despite advances in treatment, metastatic breast cancer (MBC) remains an incurable disease, with a 5-year survival rate of 25% and a median overall survival (OS) of 3 years (3).

The current management of breast cancer is primarily determined by the human epidermal growth factor receptor-2 (HER2) and hormone-receptor (HR) status (4, 5). More than two-third of breast cancers are HR-positive/HER2-negative, and endocrine therapy (ET) represents a major treatment option for these patients (6, 7). The clinical profile of these drugs, with their high efficacy and tolerability (8) helped their wide adoption (6). Endocrine therapy comprises different classes of drugs including the selective estrogen receptor modulators (SERMs) like tamoxifen, the luteinizing hormone-releasing hormone (LHRH) agonists like leuprolide, goserelin and triptorelin, the AI like letrozole, anastrozole and exemestane, the CDK4/6 inhibitors (CDK4/6i) like palbociclib, ribociclib and abemaciclib, and the selective estrogen-receptor degraders (SERDs) like fulvestrant and the more recently introduced oral agents (9). Though SERMS are widely used therapy for patient with breast cancer, its efficacy is limited and almost 25% of patients with primary and advanced-stage disease develop resistance during the course of their treatment (10, 11).

The administration of fulvestrant as bilateral intramuscular injections in a suspension of castor oil on a monthly basis is required due to its limited oral bioavailability; pain at the injection sites, can occasionally be an issue and pose challenges when considering its use in adjuvant settings where prolonged hormonal therapy lasting 5-10 years may be necessary. Additionally, the monthly injections lead to a notable peak and prolonged trough in the drug concentration within the body, which may result in suboptimal degradation of estrogen receptors (ER) (12).

In this review, we shed light on this class of molecules to explore previous, current, and future clinical uses.

## 2 The mechanism of action

**Figure 1:**
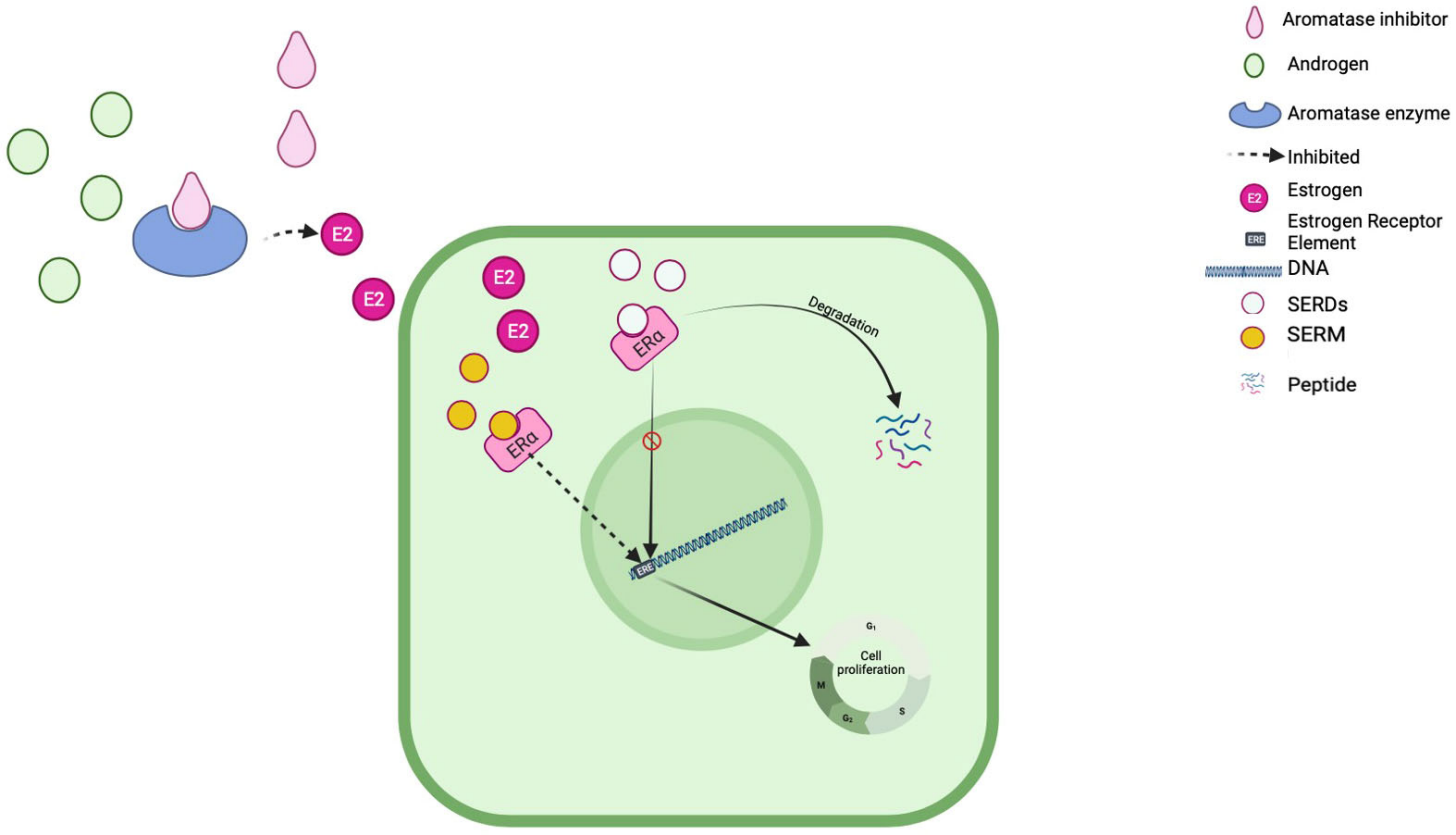
Mechanism of action.

The primary goal of endocrine therapy for metastatic HR-positive breast cancer is to aggressively counteract the impact of estrogen on cancer cells. This is accomplished through a variety of potent strategies, including the profound reduction of estrogen levels throughout the body, which serves as the formidable mechanism behind the remarkable effectiveness of AI (13). An alternative tactic involves diminishing the binding between estrogen and its receptor, exemplifying the dynamic mechanism by which SERMs exert their influence (14). Lastly, ET can reduce the number of estrogen receptors in cancer cells by terminally blocking the receptor leading to its degradation and decrease the number of the receptors, which is how our drugs of interest, SERDs exert their action (15, 16). A visual representation of the mechanism(s) by which these three drug groups work is shown in **Figure 1**.

## 3 History

The evolution and exploration of SERDs can be traced back to the 1990s. However, due to limited effectiveness and significant toxicity, the initial program was discontinued (17). Undeterred, researchers persevered, leading to the development of fulvestrant in the early 2000s. In 2002, fulvestrant received the Food and Drug Administration (FDA) approval for the treatment of advanced breast cancer (18). Subsequently, numerous clinical trials were conducted to assess the efficacy and safety of fulvestrant across various settings and phases of breast cancer therapy (19–21). As a result, in 2017, fulvestrant was granted approval in the frontline therapy for postmenopausal patients with advanced HR-positive breast cancer (22). More recently, the SOLAR-1 trial revealed that the combination of fulvestrant and alpelisib exhibited enhanced efficacy when compared to fulvestrant alone (23). This superiority was observed specifically in tumors with PI3K mutations that had experienced progression after prior endocrine therapy. Newer SERDs have shown promising results in preclinical studies and clinical trials, and their greater selectivity and potency, compared to their predecessors, indicating their potential to overcome resistance to ET (24, 25).

## 4 Candidates for SERDs

To be qualified for treatment with SERDs, patients must meet specific clinical features and their tumor should hold some molecular characteristics. The primary requirement is having a HR-positive breast cancer, along with developing resistance to other ET such AIs and SERMs (26). Additionally, patients should be in a postmenopausal state, since premenopausal women typically do not experience estrogen level changes affected by SERDs (23). It is generally advised to avoid prior exposure to SERDs, although in some cases, patients previously treated with fulvestrant may still be considered for other SERDs, although this is not commonly recommended (23).

## 5 SERDs in clinical trials

### 5.1 Fulvestrant

Fulvestrant is a synthetic steroid and a derivative of estradiol with an alkyl-sulfinyl moiety added to the endogenous estrogen receptor ligand (27). Unlike tamoxifen, it has pure anti-estrogenic effects and no apparent agonistic effects (14). It binds competitively to the estrogen receptor with a 100 times greater affinity than tamoxifen and causes downregulation of the receptor protein, which ultimately leads to complete interruption of estrogen-sensitive gene transcription (28, 29). This unique mechanism of action has demonstrated a clinical benefit rate (CBR) of 69% in postmenopausal women with tamoxifen-resistant breast cancer (30). In 2002, fulvestrant 250 mg was approved by the U.S. FDA for the treatment of ER-positive metastatic breast cancer in postmenopausal women with disease progression after antiestrogen therapy (either AI or tamoxifen) (31). The recommended dose was later revised to 500 mg after the demonstration of improved both progression-free survival (PFS) and OS, without increased toxicity versus fulvestrant 250 mg in the (CONFIRM) randomized, double-blind, phase III trial (32). Additionally, the randomized phase III FALCON trial, found that fulvestrant when given at 500 mg dose was more effective than anastrozole (33).

Fulvestrant was given a boost when it was approved to be used in combination with palbociclib, a CDK 4/6 inhibitor, in pre- and postmenopausal women to treat breast cancer progressing after ET (24). Consequently, fulvestrant in combination with ribociclib and abemaciclib have each been approved for HR-positive/HER2-negative MBC following the results of randomized Phase III studies (MONALEESA-3 and MONARCH-2), respectively; both showed improved PFS and OS (34, 35).

Although fulvestrant was generally tolerated as intramuscular injections once a month, its most common side effects were mild injection-site reactions, vasodilation, and hot flushes (36). Other well-known adverse events were asthenia, headache, gastrointestinal disturbances, urinary tract infections, and rashes (37). Moreover, recent studies have shown that fulvestrant 500 mg does not cause maximal ER downregulation *in vivo*, thus, a further increase in dose would increase the efficacy. However, that would require multiple injections each time which is less tolerable (14). In conclusion, the poor pharmacokinetic properties of fulvestrant and its injection-only administration route have directed the research community to find new oral SERDs with better pharmacokinetic properties and higher efficacy that could improve the clinical outcome (38, 39).

### 5.2 The new generation oral SERDs

The new generation oral SERDs are non-steroidal molecules that have an ER binding motif and a side chain with antiestrogenic and ER degrading activities. This side chain is either an acrylic acid or an amino acid (7). These drugs bind to the estrogen receptor and increase its hydrophobicity and instability, leading to its downregulation (14). The first developed SERD with an acrylic acid side chain was GW5638 in 1994 (40). However, this molecule and other oral SERDs with an acrylic acid side chain, including GDC-0810, AZD9496, and LSZ102 have all been discontinued as they did not show comparable or higher efficacy compared to fulvestrant (7, 14). On the other hand, new oral SERDs with basic side chains have achieved maximal ER degradation in multiple cell lines (7), in contrast to SERDs with acrylic acid side chains that do not degrade ER equally. These new SERDs have demonstrated potent activity against wild-type and mutant ER breast cancer and have reached phase III clinical trials (14).

#### 5.2.1 Giredestrant

Giredestrant (GDC9545) was designed to overcome the poor clinical performance of the previous drugs like GDC-0810and GDC0927 (7, 8). Giredestrant efficacy has led to its evaluation in both early- and advanced-stage breast cancer in several clinical trials (8, 41). It has shown antitumor activity as a single-agent with tolerable side effect profile (42, 43). In early-stage disease, the phase II coopERA study had demonstrated superior efficacy of giredestrant over aromatase inhibitors in terms of Ki67 (a proliferation biomarker) suppression in ER+ breast cancer (8, 44). In the phase II acelERA trial, giredestrant was tested in metastatic breast cancer and showed a non-statistically significant improvement in PFS; 5.6 vs 5.4 months (HR, 0.81; 95% CI, 0.60-1.10; P = 0.1757) As a result, the trial did not meet its primary endpoint. However, the benefit was more evident in patients with *ESR1* mutation with 1.8 months difference in the median PFS (HR, 0.60; 95% CI, 0.35-1.03; P = .0610) (8, 45). The drug is still being evaluated in combination with CDK 4/6 inhibitors in the double-blind randomized persevERA trial in patients with ER+/HER2- locally advanced or metastatic breast cancer (6, 7), The LidERA Phase III multicentric trial, involving 4200 patients with early ER+/HER2- breast cancer, aims to assess the efficacy and safety of adjuvant giredestrant compared to physician’s choice of adjuvant endocrine monotherapy as definitive treatment, providing crucial insights into the optimal therapeutic approach for this patient population (46). **Table 1** shows summary of clinical trial of giredestrant.

**Table 1:** Summary of clinical trial of Giredestrant.

Trial [Reference]	Masking	Other agents	Population (Sample Size)	Primary endpoint	Results
coopERA (36)	No masking	neoadjuvant window-of-opportunity phase, giredestrant 30 mg oral daily or anastrazole 1 mg QD then randomized to giredestrant or anastrazole for four 28-day cycles with 125 mg PO A Palbociclib on Days 1–21 for 16 weeks	Untreated early breast cancer postmenopausal women with ER+/HER2- breast cancer (n=221)	Change in Ki67 Scores from Baseline to Week 2	Giredestrant Ki-67 reduction 81% vs anastrozole 74%
acelERA (37)	No masking	giredestrant vs physician’s choice of ET (fulvestrant or aromatase inhibitor	Previously treated ER+/HER2- locally advanced or metastatic breast cancer. (n=303)	PFS	Median PFS 5.6 months Giredestrant arm, 5.4 months Fulvestrant/AI arm (HR, 0.81; 95% CI, 0.60-1.10; P = .1757) In ESR1 mutant cohort: Median PFS:5.3 months vs 3.5 months (HR, 0.60; 95% CI, 0.35-1.03; P = .0610)
perevERA (49)	Double masking	Giredestrant plus palbocilib vs Letrozole plus Palbociclib	ER+/HER2- locally advanced or metastatic breast cancer. (n=978)	PFS	–
lidERA (46)	none	Giredestrant vs ET	Patients with early ER+/HER2- breast cancer underwent definitive treatment (n=4200)	Evaluating the Efficacy and Safety of Adjuvant Giredestrant Compared With Physician’s Choice of Adjuvant Endocrine Monotherapy	

PFS, Progressive Free Survival; ER, Estrogen Receptor; HER2, human epidermal growth factor receptor-2; LHRH, Luteinizing Hormone-Releasing Hormone; HR, Hazzard Ratio. “-” means not available.

#### 5.2.2 Amcenestrant

Amcenestrant, also known as SAR439859, is a nonsteroidal SERD (8) that exhibits both acidic and basic properties and had demonstrated superior antagonism and degradation of estrogen receptors compared to other SERDs (47, 48). The AMEERA-1 and AMEERA-2 trials investigated the safety and efficacy of amcenestrant in postmenopausal women with advanced-stage breast cancer, particularly in heavily pre-treated patients (7). Amcenestrant has displayed excellent tolerability, with no dose-limiting toxicities. The trial has reported an objective response rate (ORR) of 10.9% and a clinical benefit rate (CBR) of 28.3%. The drug demonstrated comparable CBR in tumors expressing *ESR1* mutations (32.1%) and those without *ESR1* mutations (36.7%). This finding, based on a study involving 58 patients with known *ESR1* status, indicates that amcenestrant exhibits efficacy across both *ESR1*-mutated and *ESR1* wild-type tumors (49, 50).

In the AMEERA-3 trial, amcenestrant 400mg was compared to the standard treatment (EOC) in patients with metastatic ER-positive HER2-negative breast cancer. These patients had previously received two lines of hormonal therapy, one line of chemotherapy in the metastatic setting and were allowed CDK4/6i. The primary outcome, PFS, was nearly the same in both treatment arms, with durations of approximately 3.6 vs. 3.7 months. However, for patients with *ESR1* mutations, amcenestrant demonstrated a numerically favorable PFS of 3.7 months compared to 2.0 months with the standard treatment (HR, 0.9 [95% CI, 0.565 to 1.435]). Notably, the oral SERDs showed activity specifically in this patient subgroup (51).

Moreover, in the phase II AMEERA-4 trial, both amcenestrant 200 mg and 400 mg exhibited Ki67 suppression and demonstrated a good safety profile. This trial compared amcenestrant with letrozole at both doses (200 mg and 400 mg) in the neoadjuvant setting for postmenopausal women with operable ER+/HER2- breast cancer, specifically targeting patients with baseline Ki67 levels of 15% or higher. However, enrollment was voluntarily stopped early as informative data supporting adjuvant development became available, leading to the absence of formal statistical comparisons (52).

In the AMEERA-5 trial, amcenestrant was compared to letrozole in combination with palbociclib as a first-line treatment for metastatic HR+/HER2- breast cancer, with PFS as the primary endpoint. Unfortunately, according to the independent data monitoring committee, the combination of amcenestrant and palbociclib did not meet the pre-specified criteria for continuation compared to the control arm, resulting in the trial being stopped (6, 51, 53).

As a consequence, the sponsor decided to terminate the phase III AMEERA-6 trial, which was designed to compare amcenestrant 200 mg with tamoxifen in the adjuvant setting. Furthermore, the sponsor made the decision to discontinue the global development of amcenestrant in August, 2022 (6, 54). **Table 2** shows summary of clinical trials of amcenestrant.

**Table 2:** Summary of clinical trials of Amcenestrant.

Trial [Reference]	Masking	Other agents	Population	Primary outcome	Results
AMEERA-1 (50)	No masking	Experimental: monotherapy partA/B: Part A Dose Escalation, Part B Dose Expansion Experimental: Amcenestrant/Palbociclib: Arm #2 Part C Dose Escalation, Part D Dose Expansion Experimental: Amcenestrant/Alpelisib: Arm #3 Part F Safety Run-In, Part G Dose Expansion Experimental: Amcenestrant/Everolimus: Arm #4 Part H Dose Escalation, Part I Dose Expansion Experimental: Amcenestrant/Abemaciclib: Arm #5 Part J Dose Escalation, Part K Dose Expansion	Postmenopausal ER+/HER2- breast cancer (n=136)	DLTs, ORR, and Adverse events	Favourable safety profile, with no safety signals of bradycardia or eye disorders. Preliminary antitumor activity was observed (ORR: 10.9% and CBR: 28.3%)
AMEERA-3 (51)	No masking	Amcenestrant 400mg was compared to standard treatment (Fulvestrant, Letrozole, Exemestane, and Tamoxifen)^#^	Postmenopausal women with HR+/HER2- breast cancer with prior ET (n=290)	PFS	PFS was numerically similar between Amcenestrant and other drugs (median PFS 3.6 vs 3.7 months)
AMEERA-4 (52)*	Single	Amcenestrant at 200 mg or 400 mg was compared to Letrozole in neoadjuvant setting	Postmenopausal women with resectable stage I-III ER+/HER2- breast cancer (n=105)	Percent change from baseline in Ki67 level at Day-15	The geometric least squares (LSM) estimate of Ki67 reduction was 75.9% for Amcenestrant 400 mg, 68.2% for Amcenestrant 200 mg, and 77.7% for letrozole.
AMEERA-5 (53)*	Quadrable masking	Amcenestrant was compared to letrozole in combination with palbociclib	ER+/HER2- advanced breast cancer with no prior systemic treatment (n=1,068)	PFS	Trial stopped
AMEERA-6 (54)^*^	Quadrable masking	Compare Amcenestrant with Tamoxifen	ER+/HER2- early breast cancer who discontinued AI due to toxicity (n=3,738)	IBCFS	Trial stopped

ER, Estrogen Receptor; HER, Human Epidermal Growth Factor Receptor; ORR, Overall Response Rate; DLT, Dose-Limiting Toxicity; CBR, Clinical Benefit Rate; PFS, Progression-Free Survival; ET, Endocrine Therapy; IBCFS, Invasive Breast Cancer-Free Survival. LSM, The geometric least squares.

Prior use of CDK4/6 inhibitors was allowed.

*Enrolment was voluntarily stopped.

#### 5.2.3 Camizestrant

Camizestrant (AZD9833) is another new non-steroidal oral SERD. Its novel structure contributed to its increased potency and facilitated a unique pattern of gene regulation (8). In preclinical patient-derived xenograft models, it promotes ER degradation and inhibits tumor cell growth, including *ESR1*-mutant cells (55). Unlike fulvestrant, it has not shown any relative dose-dependent resistance in the *ESR1*-mutant cells (8).

##### 5.2.3.1 Background of ongoing studies

In the phase I SERENA-1 trial, camizestrant monotherapy was assessed at different doses ranging from 25 mg to 450 mg in women with ER+/HER2- breast cancer. The patients were pre-treated with more than one line of endocrine therapy and less than two lines of chemotherapy (40). 46% of patients had detectable *ESR1* mutation in the baseline ctDNA samples. The results demonstrated good efficacy and dose-dependent safety profile. Evidence of clinical benefit was observed at all dose levels (56). The overall response rate (ORR) was 16.3%, and the clinical benefit rate (CBR) was 42.3%. In patients with *ESR1* mutations, 50% had a partial response or stable disease at 24 weeks of therapy (8, 47). Camizestrant-related side effects were mostly grade 1 and 2 visual and gastrointestinal disturbances, asymptomatic bradycardia, and fatigue. Dose-limiting toxicities were only observed at 300 mg and 450 mg doses (48). In the other two parts of the trial (C/D), camizestrant 75 mg was evaluated in combination with palbociclib. The results that were published in 2021 demonstrated efficacy and tolerability. None of the patients experienced camizestrant-related grade ≥3 toxicities. Furthermore, patients with prior heavy endocrine treatment had a clinical benefit rate of 28% (57). Due to the previous encouraging findings, several ongoing trials are evaluating camizestrant in multiple settings. The SERENA-3 trial is an ongoing randomized open-label phase II study that examines the biological effects of 75-150 mg camizestrant given once daily in early-stage treatment-naïve ER+/HER2- breast cancer (58). Moreover, the SERENA-4 and SERENA-6 trials are phase III randomized double-blind ongoing studies. The primary endpoint of the SERENA-4 trial is the PFS in camizestrant plus palbociclib versus anastrozole plus palbociclib in *de novo* or recurrent ER+/HER2- breast cancer (59). In the SERENA-6 trial, camizestrant is being compared to aromatase inhibitors when combined with palbociclib or abemaciclib in ER+/HER2- metastatic breast cancer (60). **Table 3** shows summary of clinical trials of camizestant.

**Table 3:** Summary of the clinical trials of Camizestrant.

Trial	Masking	Other agents	Population	Primary outcome	Results
SERENA-1 (57)	No masking	Parts A/B: use different doses of camizestrant Parts C/D examine camizestrant 75 mg in combination with Palbociclib 75 mg	Women with ER+/HER2- Advanced Breast Cancer (n=403)	Dose-limiting toxicity (DLT) and adverse events	DLT at 300 mg and 450 mg: G1: Visual disturbances, bradycardia, nausea, fatigue, dizziness, vomiting, asthenia
SERENA-2 (62)	No masking	Arm A: Camizestrant at 75 mg or 150 mg daily Arm B: Fulvestrant at 500 mg by intramuscular injection every 4 weeks	Postmenopausal women with advanced ER+/HER2- breast cancer (n=240)	PFS	Median PFS: Camizestrant 75 mg: 7.2 months Camizestrant 150mg: 7.7 months Fulvestrant: 3.7 months
SERENA-3 (58)	No masking	In Stage-1, randomized 1:1 to receive either 75 mg or 150 mg oral Camizestrant daily for 5-7 days, followed by a minimum 5-day pre-surgery washout Stage-2 will include participants across up to 3 treatment groups. Stage 3 will include two treatment groups.	ER+/HER2- primary breast cancer (n=132)	Change from baseline in ER expression	–
SERENA-4 (59)	Randomized, double-blind	(a) Camizestrant 75 mg, once daily, Palbociclib 125 mg, once daily for 21 days followed by 7 days off treatment (b) Anastrozole (1 mg, once daily), palbociclib (same as active arm),	ER+/HER2- advanced/metastatic breast cancer with no prior treatment (n=1342)	PFS	–
SERENA-6 (60)	Randomized, double-blind	Step1:CDK4/6i (palbociclib or abemaciclib) with AI (letrozole or anastrozole) Step 2 (upon detection of ESR1 mut without clinical or radiological disease progression Randomized into 2 arms: Arm A: continue with same AI Arm B: Switch to camizestrant	ER+/HER2- breast cancer on current 1 line SOC with detectable ESR1 mutation. (n=302)	PFS	–
CAMBRIA-1 (67)	Randomized, Open label	Arm A: Continue standard ET of investigator’s choice (aromatase inhibitors [AI; exemestane, letrozole, anastrozole] or tamoxifen) Arm B: Camizestrant	patients with ER+/HER2 - early breast cancer with intermediate or high risk for disease recurrence who completed definitive locoregional therapy (with or without chemotherapy) and standard adjuvant endocrine therapy (ET) for at least 2 years and up to 5 years. The planned duration of treatment in either arm of the study is 60 months (n=4300)	Invasive breast cancer-free survival (IBCFS)	
CAMBRIA-2 (68)	Randomized phase III	Arm A: Camizestrant Arm B: Standard Endocrine Therapy (Aromatase Inhibitor or Tamoxifen)	Patients With ER+/HER2- Early Breast Cancer and an Intermediate-High or High Risk of Recurrence Who Have Completed Definitive Locoregional Treatment and Have No Evidence of Disease (n=5500)	Invasive breast cancer-free survival (IBCFS) and main secondary endpoints include Invasive disease-free survival (IDFS), Distant relapse-free survival (DRFS), Overall survival (OS), Safety and Clinical Outcome Assessments (COAs).	

ER, Estrogen Receptor; HER, Human Epidermal Growth Factor Receptor; DLT, Dose-Limiting Toxicity; G1, Grade 1; PFS, Progression-Free Survival; SOC, standard-of-care; ESR1, Estrogen Receptor Gene 1. “-” means not available.

##### 5.2.3.2 SERENA-2 trial (camizestrant vs fulvestrant)

The SERENA-2 trial (61), a randomized, parallel-group, multicenter phase II study, comparing the safety and efficacy of 3 different doses of camizestrant with fulvestrant 500 mg in the treatment of postmenopausal women with ER+/HER2- advanced-stage breast cancer with disease recurrence or progression after at least one line of endocrine therapy (55). Furthermore, the study included patients with no more than one line of chemotherapy and no prior fulvestrant treatment while prior treatment with CDK4/6i was permitted (55). Eligible women who have met the inclusion criteria were then randomized into 4 intervention arms 1:1:1:1. The interventions were camizestrant 75 mg, 150 mg, 300 mg, or fulvestrant 500 mg (55). The primary endpoint was PFS as assessed by response evaluation criteria in solid tumors (RECIST) from the date of randomization to the date of disease progression or death (53). Other secondary outcomes measured were the objective response rate (ORR), duration of response (DoR), OS, clinical benefit rate at 24 weeks, and effect on health-related quality of life (HRQoL). In addition to other pharmacokinetic and pharmacodynamic effects of Camizestrant (47, 55, 62).

Results from the trial were presented at the 2022 San Antonio Breast Cancer Symposium (SABCS). Camizestrant at 75 mg and 150 mg doses have shown statistically significant improvement in PFS compared to fulvestrant. In the overall population, camizestrant at 75 mg and 150mg reduced the risk of disease progression by 42%, and 33%, respectively (63). The median PFS on camizestrant 75 mg was 7.2 months and 7.7 months for camizestrant 150 mg, but 3.7 months for fulvestrant. Outcomes were better in patients with *ESR1*-mutated tumors; camizestrant reduced the risk of death or disease progression by 67% at 75 mg and by 45% at 150 mg dose, compared to fulvestrant. Improvement in PFS was also demonstrated in patients previously treated with CDK4/6i and patients with lung and/or liver metastases (57). The most common treatment-related adverse events were photopsia (12.2%, 24.7%, 35.0%, and 0%) and bradycardia (5.4%, 26.0%, 40.0%, and 0%), for 75 mg, 150 mg, 300 mg camizestrant or fulvestrant (57).

#### 5.2.4 Elacestrant

Elacestrant (RAD1901) is a novel oral SERD with a basic side chain first reported in 2015 (7). It was developed by Radius health for use in ER+ breast cancer (8). Elacestrant exhibits significant dose-dependent antitumor activity in preclinical models (64). In a phase I study, elacestrant at a dose of 400 mg once daily, demonstrated single-agent activity with confirmed partial responses in ER+ heavily pre-treated metastatic breast cancer patients with an acceptable safety profile (65). These data provided the rationale for the phase III EMERALD study comparing the efficacy and safety of elacestrant versus standard-of-care endocrine treatment (fulvestrant or AI) in patients with ER+, HER2− advanced breast cancer (66). In this multicenter study, 478 patients with ER-positive, HER2-negative advanced or metastatic breast cancer were enrolled; 228 (48%) of them had *ESR1*-mutated tumors (60). The study population had disease progression following one or two prior lines of endocrine therapy, including at least one line containing a CDK4/6i. As such, this is the only trial in the second line and beyond were all participants had previous exposure to CDK4/6i in metastatic setting Additionally, one prior line of chemotherapy in the advanced or metastatic setting were allowed (60). Patients were randomized to receive the investigator’s choice of endocrine therapy (including fulvestrant or an AI) or elacestrant 345 mg orally once daily (60). Stratification factors were *ESR1* mutation status, prior treatment with fulvestrant and presence of visceral metastasis. *ESR1* mutational status was determined by analyzing blood circulating tumor deoxyribonucleic acid (ctDNA) using the Guardant360 CDx assay (60). PFS was the primary endpoint for efficacy assessment and showed statistically significant improvement with elacestrant; 30% reduction in risk of disease progression and death, and even greater benefit in *ESR1*-mutant with 45% risk reduction compared to standard of care (SOC) (60).

The duration of prior CDK4/6i had a positive impact on PFS when treated with elacestrant, whereas no such association was observed with the SOC; the median PFS for elacestrant group treated with 12 months or longer of CDK4/6i was 3.8 months, but higher (5.5 months) for those treated for 18 months or longer, compared to 1.9 months and 3.3 months, respectively in SOC group. In the *ESR1*-mutant group, the median PFS with at least 12 months of prior exposure to CDK4/6i approached 8.6 months with elacestrant compared to only 2.1 months with the SOC; a 53% reduction in the risk for disease progression or death, making the *ESR1* mutation and duration on previous CDK4/6i as predictive markers for response. The most frequently reported adverse events (occurring in at least 10% of patients) during the study included musculoskeletal pain, fatigue, nausea, vomiting, decreased appetite, diarrhea, headache, constipation, abdominal pain, hot flushes, and dyspepsia. Laboratory abnormalities include increased cholesterol and triglyceride levels, elevated liver enzymes (AST and ALT), anemia, decreased sodium levels and mild renal impairment (60).

#### 5.2.5 Imlunestrant

Imlunestrant represents an innovative orally bioavailable SERD characterized by its pure antagonistic attributes, leading to continuous inhibition of estrogen receptor (ER)-dependent gene transcription and cell growth.

The EMBER trial, a multicenter, open-label phase Ia/b dose-escalation/expansion trial, included patients with ER+ advanced breast cancer (prior endocrine therapy sensitivity; ≤3 prior therapies for advanced breast cancer). The phase Ia/b of the EMBER trial is assessing the efficacy of imlunestrant alone and in combination with other agents for ER+/HER2- advanced breast cancer. At the recommended phase 2 dose (RP2D) of 400 mg once daily (n= 69), the most common all-grade treatment-emergent adverse events (TEAEs) were nausea (33.3%), fatigue (27.5%), and diarrhea (23.2%). Across all doses, the incidence of treatment-related grade 3 adverse events was low (3.6%). No patient discontinued treatment due to a TEAE. In evaluable advanced breast cancer patients, the objective response rate (ORR) was 8.0% (6/75), and the clinical benefit rate (CBR) was 40.4% (42/104). Clinical benefit was observed regardless of baseline *ESR1* mutation status as determined by circulating tumor DNA sequencing (69).

Imlunestrant in combination with abemaciclib ± AI demonstrated acceptable safety and tolerability, ORR was 36% (10/28) for imlunestrant and abemaciclib vs 44% (15/34) for imlunestrant and abemaciclib plus AI. These findings suggest no additional toxicity of imlunestrant when administered with abemaciclib, along with comparable clinical benefit to that observed in MONARCH 2 (70).

Updated data on imlunestrant ± everolimus or alpelisib arms at ESMO 2023 reveals the following tumor response rates: 8% (6/76) for imlunestrant monotherapy (114 patients), 21% (6/28) for imlunestrant + everolimus (42 patients), and 58% (7/12) for imlunestrant + alpelisib (21 patients). Imlunestrant, either alone or in combination with everolimus or alpelisib, demonstrated strong efficacy in pre-treated ER+, HER2- advanced breast cancer. Toxicities were in line with the known safety profiles of alpelisib and everolimus (71).

EMBER-3 is a randomized phase 3 clinical trial investigating the efficacy of imlunestrant compared to the investigator’s selected endocrine therapy, which includes either fulvestrant or exemestane. The study focuses on patients diagnosed with ER-positive, HER 2-negative, locally advanced, or metastatic breast cancer who have undergone prior treatment with endocrine-based therapy. The primary endpoint of the trial is PFS in both the intention-to-treat (ITT) population and in patients with *ESR1* mutation (72).

EMBER4 is a randomized, open-label, global phase 3 study comparing imlunestrant versus physicians’ choice of ET, in patients who are at an increased risk of recurrence based on clinico-pathological features and who have received 2 to 5 years of standard adjuvant ET. Approximately 6,000 patients will be randomized 1:1 to receive imlunestrant (400 mg daily) for 5 years or physicians’ choice of adjuvant ET (tamoxifen or an aromatase inhibitor). Study treatment duration is 5 years with Invasive Disease-Free Survival (IDFS) as primary outcome (73).

#### 5.2.6 Palazestrant

OP-1250 is an innovative, orally available medication that acts as a complete estrogen receptor (ER) antagonist and selective ER degrader. OP-1250 entirely prevents estrogen from activating transcriptional activity and lacks any agonist effects on the ER. This drug exhibits strong binding affinity, ER degradation, and antiproliferative properties in ER-positive breast cancer models, often matching or exceeding the performance of other comparable treatments.

OP-1250 offers superior pharmacokinetic benefits with the ability to cross the blood-brain barrier. It has demonstrated strong efficacy in wild-type and ESR1-mutant breast cancer xenograft models. OP-1250 works well in combination with cyclin-dependent kinase 4 and 6 inhibitors in preclinical studies, leading to significant tumor shrinkage in intracranial breast cancer models and extending the lifespan of the test subjects.

international, multicenter phase III clinical trial OPERA-01 is designed to evaluate the safety and efficacy of palazestrant (OP-1250) as a monotherapy compared to standard endocrine treatments: either fulvestrant or an aromatase inhibitor (anastrozole, letrozole, or exemestane). It is an open-label, randomized, active-controlled study.

The trial is recruiting adult patients with advanced or metastatic breast cancer that is hormone receptor-positive (ER+) and HER2-negative. These patients must have experienced disease progression or relapse after one or two previous lines of standard-of-care endocrine therapy for metastatic breast cancer. One of these lines must have involved a combination of endocrine therapy and a CDK 4/6 inhibitor.

The initial dose-selection phase of the trial involves approximately 120 participants, who will be randomly assigned to one of two doses of palazestrant or to the standard endocrine therapy. Subsequently, around 390 participants will be randomly assigned to either the chosen dose of palazestrant or to the standard endocrine therapy (74).

Research is still ongoing for other agents such as borestrant, rintodestrant, and taragarestrant.

## 6 Discussion

The introduction of CDK4/6i, initially in the metastatic setting and more recently in the adjuvant setting, have made significant progress in the treatment of patients with HER2-negative, HR-positive breast cancer. However, researchers were hoping to find a more effective hormonal therapy that can beat both AI and fulvestrant when used alone or in combination with CDK4/6i. The intramuscular formulation of fulvestrant have highlighted the need for alternative solutions, too.

Furthermore, the growing understanding of the role of *ESR1* mutations in endocrine therapy resistance for SERMs and AIs has intensified the pursuit of oral SERDs as a potential solution. In addition to addressing these challenges, oral SERDs offer the added advantage of enhancing the efficacy of agents that target other molecules involved in cross-talks with ER pathways. This multifaceted approach positions oral SERDs as promising candidates to overcome these limitations and optimize treatment outcomes in endocrine therapy-resistant breast cancer.

Results from the phase III EMERALD trial indicate that elacestrant offers an improvement in 12-month PFS compared to standard of care therapy for patients with ER+ metastatic breast cancer who have experienced disease progression on prior endocrine therapy. Furthermore, the clinical benefit of elacestrant was particularly significant in patients with *ESR1* mutations (66). These findings are highly promising and suggest a potential paradigm shift towards the use of oral SERDs as an effective treatment option for ER+ breast cancer.

Camizestrant also has shown promising efficacy in treating ESR1 mutations in breast cancer, as evidenced by its significant improvement in progression-free survival (PFS) compared to fulvestrant. At doses of 75 mg and 150 mg, camizestrant reduced the risk of disease progression by 42% and 33%, respectively, in the overall population. For patients with ESR1-mutated tumors, camizestrant at these doses led to even greater reductions in the risk of death or disease progression—67% at 75 mg and 45% at 150 mg—compared to fulvestrant. These results highlight camizestrant’s potential as a valuable therapeutic option for patients with ESR1 mutations, offering a meaningful advancement in breast cancer treatment.

Despite the progress made in understanding the role of *ESR1* mutations and the potential efficacy of SERDs, there are still important questions need to be to addressed in optimizing endocrine therapy. While *ESR1* mutations are associated with increased likelihood of response to SERDs due to tumor dependence on ER-mediated signaling, this association is not always reliable or perfect in clinical settings. In the EMERALD trial, approximately half of the patients demonstrated intrinsic resistance to ET, regardless of the treatment arm, underscoring the fact that some patients may not benefit from this approach. Moreover, the presence of polyclonal resistance poses an additional challenge, as tumors with *ESR1* mutations often have subclones harboring concurrent genomic alterations that could confer resistance through ER-pathway-independent mechanisms (66).

Characterizing metastatic tumors solely based on ER positivity or *ESR1* status is insufficient, and there is a need for improved methodologies to select patients who remain endocrine sensitive after CDK4/6i treatment. This may involve the use of genomic profiling panels or the identification of novel biomarkers that can more accurately characterize tumors susceptible to endocrine therapy. Advancements in this area of research can significantly impact patient care by identifying the most appropriate treatment strategies.

Two oral SERDs, namely amcenestrant and giredestrant, failed to meet their primary endpoints in separate clinical trials. The AMEERA-3 trial included patients who had previously failed CDK4/6i and two lines of hormonal therapy, while the *ESR1* mutation status was not assessed (51). Speculation arises that amcenestrant might still demonstrate superiority over the control arm if only patients with *ESR1*-mutated tumors were enrolled. Similarly, the acelERA trial that compared giredestrant with fulvestrant or aromatase inhibitor, did not achieve its primary endpoint of superior PFS for the study drug. However, subgroup analysis of patients with baseline *ESR1* mutations indicated 1.8 months difference in the median PFS (HR, 0.60; 95% CI, 0.35-1.03; p=0.0610) (8, 45). Notably, giredestrant exhibited positive outcomes in reducing Ki67 expression and inducing complete cell cycle arrest when used as neoadjuvant therapy in previously untreated patients with hormone receptor-positive, HER2-negative early breast cancer in the coopERA study (8, 44).

Concerns regarding the potential effects of oral SERDs on the cardiac conducting system and cornea were raised prior to the presentation of the EMERALD trial results. Earlier trials of camizestrant and giredestrant reported cases of bradycardia, and QT prolongation was observed with camizestrant and amcenestrant. Ocular toxicity was primarily associated with camizestrant and giredestrant. It is worth noting that the cardiac conducting system and cornea do not express ER. In the past, such toxicities were rarely observed in patients treated with other antiestrogen agents, including tamoxifen, AI, and fulvestrant. This suggests that the ocular or cardiac toxicities observed with specific oral SERDs are unlikely to be solely caused by on-target effects against ER. Reassuringly, the EMERALD trial did not report any ocular or cardiac toxicities (66). Nonetheless, the underlying mechanisms behind the occurrence of these side effects with certain oral SERDs while others do not cause them remain the subject of investigation and scrutiny.

Accordingly, in January, 2023, elacestrant was approved by the Food and Drug Administration (FDA) for postmenopausal women or adult men with ER-positive, HER2-negative, *ESR1*-mutated advanced or metastatic breast cancer with disease progression following at least one line of endocrine therapy. Additionally, Guardant360 CDx assay was approved as a companion diagnostic device to identify patients for treatment with elacestrant.

## 7 Conclusions

Endocrine therapy, including SRDs and aromatase inhibitors, play a vital role in the effective treatment of breast cancer; both in early- and advanced-stage disease. The recently introduced oral SERDs are promising players, alone or in combination with other agents like the CDK4/6i. The increase utilization of genomic profiling and novel biomarkers, may accurately characterize tumor subtypes that are more susceptible to specific endocrine therapy.

## Author contributions

BS: Data curation, Validation, Writing – original draft, Writing – review & editing. AA: Data curation, Validation, Writing – original draft, Writing – review & editing. HH: Data curation, Validation, Writing – original draft, Writing – review & editing. HA-R: Conceptualization, Supervision, Writing – original draft, Writing – review & editing.
